# 564. Nocardiosis in Portugal: a single-center experience

**DOI:** 10.1093/ofid/ofad500.633

**Published:** 2023-11-27

**Authors:** Bruno Besteiro, Daniel Coutinho, Joana Fragoso, Cristovão Figueiredo, Sofia Nunes, Carlos Azevedo, Tiago Teixeira, Aurelia Selaru, Angelina Lameirão, Luis Malheiro

**Affiliations:** Centro Hospitalar e Universitário de São João, Oporto, Porto, Portugal; Infectious Diseases Department, Centro Hospitalar de Vila Nova de Gaia Espinho, Vila Nova de Gaia, Portugal - Oporto (Portugal), Oporto, Porto, Portugal; Infectious Diseases Department, Centro Hospitalar de Vila Nova de Gaia Espinho, Vila Nova de Gaia, Portugal - Oporto (Portugal),, Oporto, Porto, Portugal; Infectious Diseases Department, Centro Hospitalar de Vila Nova de Gaia Espinho, Vila Nova de Gaia, Portugal - Oporto (Portugal),, Oporto, Porto, Portugal; Infectious Diseases Department, Centro Hospitalar de Vila Nova de Gaia Espinho, Vila Nova de Gaia, Portugal - Oporto (Portugal),, Oporto, Porto, Portugal; Infectious Diseases Department, Centro Hospitalar de Vila Nova de Gaia Espinho, Vila Nova de Gaia, Portugal - Oporto (Portugal),, Oporto, Porto, Portugal; Infectious Diseases Department, Centro Hospitalar de Vila Nova de Gaia Espinho, Vila Nova de Gaia, Portugal - Oporto (Portugal),, Oporto, Porto, Portugal; Microbiology Department, Centro Hospitalar de Vila Nova de Gaia Espinho, Vila Nova de Gaia, Portugal - Oporto (Portugal), Oporto, Porto, Portugal; Microbiology Department, Centro Hospitalar de Vila Nova de Gaia Espinho, Vila Nova de Gaia, Portugal - Oporto (Portugal), Oporto, Porto, Portugal; Infectious Diseases Department, Centro Hospitalar de Vila Nova de Gaia Espinho, Vila Nova de Gaia, Portugal - Oporto (Portugal), Oporto, Porto, Portugal

## Abstract

**Background:**

Nocardiosis is a rare opportunistic infection with increasing incidence. Given its rarity, data on the prognosis and distribution are scarce and essential.

**Methods:**

Retrospective study reviewing all nocardiosis cases diagnosed at our tertiary care hospital from January 2019 to January 2023.

**Results:**

A positive *Nocardia spp.* culture was identified in 20 patients. Nocardia isolation was considered colonization in 2 patients, but the other 18 cases were considered as disease, 6 (33.3%) of which were classified as disseminated nocardiosis (DN). The mean

age was 64.0±14.05 years-old and 75% were males. One or more immunosuppressive conditions were identified in 70% of patients, including diabetes mellitus (n=9), active solid tumors (n=3), autoimmune diseases (n=3) and HIV infection with CD4+ < 200 cel/uL (n=2). Among the immunocompromised patients, 28,6% were being treated with high-dose corticosteroid therapy. The lung was the most common site of infection (85%) and most cultures were initially retrieved for lower respiratory tract samples (85%) evaluated for mycobacteria in modified Middlebrook 7H9 Broth. Lymphopenia was observed in 38.9% of patients.The most frequently isolated species were *N.nova/africana* (n=7), *N.cyriacigeorgica* (n=4) and *N.pseudobrasiliensis* (n=3). *N.cyriacigeorgica* was identified in the two cases considered colonization. Most patients (94.4%) were treated with antimicrobials, 50.0% in monotherapy and 44.4% in combination therapy. The most frequently prescribed antibiotics were co-trimoxazole (94.4%), imipenem (22.2%) and linezolid (16.7%). Selected antimicrobial agents were generally effective, with linezolid and co-trimoxazole having the highest susceptibility rates (100% susceptibility). The median [IQR] duration of treatment was 238 [45–720] and 170 [5–360] days for localized and DN, respectively. The overall one- year case fatality was 25% and was higher in patients with DN (80%).

**Conclusion:**

Nocardiosis is an uncommon but emerging disease which can occur both in immunocompetent and immunocompromised patients. The present study reports a case series on Nocardiosis from Portugal and provides important information of this disease. As the

outcomes are poor, early recognition and prompt treatment are essential to improve the outcome.

**Disclosures:**

**All Authors**: No reported disclosures

